# Regulating Methylation at H3K27: A Trick or Treat for Cancer Cell Plasticity

**DOI:** 10.3390/cancers12102792

**Published:** 2020-09-29

**Authors:** Provas Das, Joseph H. Taube

**Affiliations:** Department of Biology, Baylor University, Waco, TX 76706, USA; Provas_Das@baylor.edu

**Keywords:** H3K27me3, EZH2, KDM6A, KDM6B, cancer cell plasticity

## Abstract

**Simple Summary:**

Regulation of gene expression is important for appropriate cell development but can also lead to inappropriate cell transformation resulting in cancer. Modification to the proteins that wrap DNA is essential for regulating gene expression. Mutations in the enzymes that modify such proteins are being discovered in many cancers. This review will cover present knowledge of these enzymes as they relate to cancer initiation and progression.

**Abstract:**

Properly timed addition and removal of histone 3 lysine 27 tri-methylation (H3K27me3) is critical for enabling proper differentiation throughout all stages of development and, likewise, can guide carcinoma cells into altered differentiation states which correspond to poor prognoses and treatment evasion. In early embryonic stages, H3K27me3 is invoked to silence genes and restrict cell fate. Not surprisingly, mutation or altered functionality in the enzymes that regulate this pathway results in aberrant methylation or demethylation that can lead to malignancy. Likewise, changes in expression or activity of these enzymes impact cellular plasticity, metastasis, and treatment evasion. This review focuses on current knowledge regarding methylation and de-methylation of H3K27 in cancer initiation and cancer cell plasticity.

## 1. Introduction

Regulation of gene expression depends on access to the genome and recruitment of trans-acting factors, both of which are processes that are impacted by the histone modification state. Modification of specific histones can be pathologically altered by aberrant “writers” that add modifications, “erasers” that remove modifications, or by the mutation of the histone structure which can prevent the residues from being modified [1,2]. Along with these modifications, alteration in the “readers” can alter the functional outcome of the modification. Tri-methylation of the 27th lysine residue on histone H3 (H3K27me3), while just one of many such modifications, plays an important role in developmentally driven gene silencing and in cancer initiation and progression. This review will cover present knowledge on the role of H3K27me3 and the enzymes which modify this residue in cancer, with a particular focus on cellular plasticity.

## 2. Histone Methylation and Demethylation: An Historical Perspective

Although largely thought of as a genetic disease, current evidence establishes that epigenetic aberrations can play profound and ubiquitous roles in cancer origin and progression [3,4,5]. Results from large-scale cancer genome sequencing projects describe that an unanticipated 50% of human cancers harbor mutations in chromatin-related proteins [6,7]. Overwhelming evidence supports the notion that specific genetic, environmental, and metabolic stimuli can disrupt the homeostatic balance in chromatin machinery, which can cause either aberrantly restrictive or permissive responses. Such stimuli can act on quiescent or premalignant cells to promote malignancy and/or tumor cells to hasten their progression and adaptation. The ubiquity of such stimuli means that epigenetic defects are involved in various aspects of cancer [8,9,10].

Histone modifications, DNA methylation, chromatin architecture, and the expression of non-coding transcripts drive the epigenetic state of a cell. The N-terminal tails of histones can undergo a variety of post-translational covalent modifications which can then impact critical DNA-dependent processes like replication, transcription, and repair [11,12]. Histone methylation is mediated by methyltransferases that catalyze the mono-, di-, or tri- methylation of specific residues. Dynamic and reversible methylation of histones plays an important role in the regulation of gene expression. Two major families of histone methyltransferases are lysine methyltransferases (KMTs) and protein arginine methyltransferases (PRMTs). Whereas methylation of some lysine residues is associated with activation of gene transcription (e.g., H3K4, H3K36, and H3K79), methylation of other residues is associated with repression of gene transcription (e.g., H3K9, H3K20, and H3K27) [13,14,15]. Some histone modifications are enriched at distinct regions (i.e., enhancer, promoter, or coding sequences) of the gene, though H3K27me3 is distributed widely across enhancers and promoters [16]. Moreover, mono-, di-, or tri-methylation of a single residue can alter the affinity of “reader” proteins to the methylated histones and may have distinct functional consequences [17].

**Figure 1:**
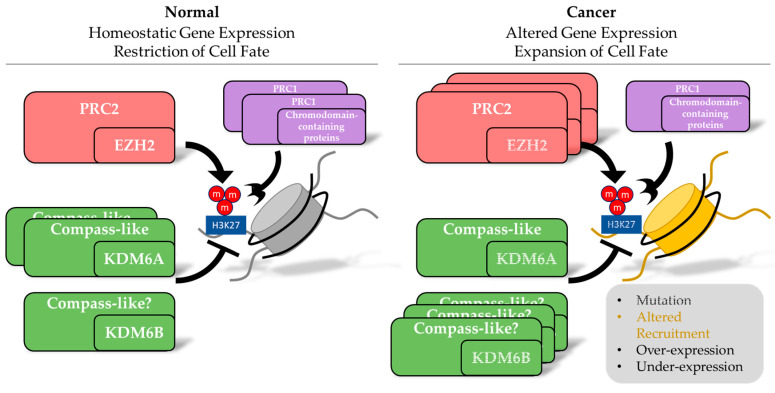
Regulated addition and removal of H3K27me3 is critical for maintaining cellular homeostasis. Functioning as members of protein complexes, the indicated enzymes possess the biochemical activity to alter the methylation state at H3K27 while chromodomain-containing proteins, among others, have the capacity to interpret the methylation state. Disruption in this balance, whether through mutations (indicated by strikethroughs), altered recruitment (indicated by change in nucleosome color), over-expression (indicated by additional boxes) or under-expression (indicated by fewer boxes), has the potential to drive and/or support tumorigenesis through the expansion of cell fate identity.

In particular, tri-methylation of the lysine at the 27th amino acid on histone H3 (H3K27me3) is a critical determinant of chromatin accessibility and gene expression. The “writer” of H3K27me3, enhancer of zeste 2 (EZH2) (Figure 1), has been known to be involved in tumor progression in an oncogenic capacity for over twenty years [18,19,20]. Detailed reviews concerning the activity and history of the H3K27me3 methyltransferases, EZH1 and EZH2, are available [21,22]. Nevertheless, the more recent uncovering of Jumonji-domain-containing proteins, which are capable of catalyzing the demethylation of H3K27me3, has revealed additional complexity surrounding H3K27me3 (Figure 1).

### 2.1. Molecular Mechanisms

The primary H3K27me3 “writer” protein, EZH2, is the enzymatic component of the polycomb repressor complex 2 (PRC2) and is capable of catalyzing methylation of non-methylated H3K27 to either a di- or tri-methylated state. The C-terminal SET domain of EZH2 exhibits the methyltransferase activity. Along with EZH2, additional proteins including EED, SUZ12, and RBBP4/RbAp48/NURF55 comprise the core of the PRC2 complex [23] (Figure 1). All of these proteins are required for the full histone methyltransferase activity of EZH2 [23,24,25]. The zinc-finger containing protein, SUZ12, and WD40 repeat protein, EED, together maintain the structural integrity of PRC2 complex and methyltransferase activity of EZH2. Additional PRC2 components that fall outside the core complex include AEP2, PCL (PHF), and JARID2 which appear to be accessory factors for PRC2 enzymatic activity. The PRC2 subcomplex, which lacks the fourth core component RBBP4/RbAp48/NURF55 can nevertheless perform substantial enzymatic activity. EZH2 is post-translationally regulated by O-linked N-acetylglucosamine (GlcNAc) transferase (OGT)-mediated O-GlcNAcylation at S75, which stabilizes EZH2 and facilitates the formation of H3K27me3 [26]. Although, EZH1 is a paralog of EZH2, the EZH1-loaded PRC2 complex has a distinct cellular function, primarily as a maintainer of the repressed chromatin state. Indeed, EZH1 is ubiquitously expressed, whereas EZH2 is expressed primarily in proliferating cells. These observations suggest that EZH1-PRC2 containing complexes might contribute in the restoration of the H3K27 methylation after histone demethylation or histone exchanges [23,27]. H3K27me3 is detected by specific binding of chromodomain-containing protein, CBX, in the polycomb repressor complex 1 (PRC1). A component of PRC1 (Ring1b) then mediates mono-ubiquitination of H2A at K119 [28]. This modification (H2AK119ub1) is required for PRC-dependent silencing and functions to inhibit RNA polymerase II elongation [29].

In contrast to other histone modifications such as phosphorylation and acetylation, methylation and especially trimethylation, have been considered irreversible because of the high thermodynamic stability of the N-CH_3_ bond [30,31,32]. Nevertheless, while lysine demethylases (KDMs) were first detected in the 1960s in rat kidney extracts, it took four decades to identify specific proteins with such activity. The first such enzyme described, Lysine-Specific Demethylase 1 (initially named LSD1 and then re-named to KDM1A), is an amine oxidase that can demethylate di- or mono-methylated H3K4 [30,31,33,34,35]. However, a second discovery uncovered a much larger class of lysine-specific demethylases which are categorized as Jumonji C (JmjC) domain-containing (JMJD) proteins. To date around 30 KDMs have been identified and characterized [36]. Based on their mechanism of action, KDMs can be classified into two families: (1) Flavin adenine dinucleotide (FAD)-dependent, which can catalyze the oxidation of a methyl amine group to generate an iminium cation, and (2) Fe (II) and 2-oxoglutarate (2OG)-dependent, which can catalyze reactions by converting the methyl group into a hydroxymethyl group, then released as formaldehyde [30,37,38,39]. The FAD-dependent family KDM1 (KDM1A and KDM1B) can only catalyze the demethylation of mono- and di-methylated lysine (Kme1 and Kme2), unlike the Fe (II) and 2OG-dependent family of KDMs (KDM 2–7), which are capable of demethylating Kme1, Kme2 and Kme3 [40,41]. A series of magnificent reviews have provided a detailed and extensive characterization of individual histone demethylases, their substrates and their roles in normal development and diseases [36,41,42,43,44].

### 2.2. Intersection with DNA Methylation

Methylation at the 5ʹ position of cytosine residues within the DNA (5mC) is an epigenetic mark that can also alter gene expression. When present in promoter regions, 5mC typically suppresses gene expression by recruiting the methyl binding proteins and modulation of transcription machinery access. 5mC is an important mechanism in normal cell development, by playing roles in regulation of gene expression, X-chromosome inactivation, genetic imprinting, and the suppression of repetitive element transcription and transposition. Aberrant 5mC may occur through hypermethylation in the promoter regions of tumor suppressor genes, resulting in silencing of that gene or hypomethylation in the promoter regions of oncogenes, activating them.

Though similar in their effect on transcriptional output, 5mC and H3K27me3 are used in distinct regions across the genome. At CpG-rich regions of the genome 5mC and H3K27me3 show little overlap in both normal and cancer cells, a phenomenon which may be explained by diminished binding of the PRC2 complex to chromatin enriched with 5mC [45,46]. However, at CpG-poor regions, silenced chromatin frequently contains both 5mC and H3K27me3 [47].

Biochemically, 5mC and H3K27me3 are intimately linked. The conversion of 5mC into an oxidized form capable of replacement through base-excision repair is accomplished by the TET family of enzymes and is dependent on O_2_, Fe(II), and 2-OG [48] through a similar mechanism to that which is used by the JmjC lysine de-methylases. Thus, levels of oxygen, iron, and metabolites can affect the stability of both 5mC and H3K27me3. Furthermore, the established 5mC reader protein, MeCP2, was recently shown to also bind to H3K27me3-enriched nucleosomes [49], illustrating conservation in the two transcription silencing mechanisms.

### 2.3. Developmental Roles

The discovery of the reader and writer protein complexes for H3K27me3 as mutants that disrupt developmental patterning in the fruit fly illustrates how important this residue is in developmental processes. In mice, loss of PRC2 components EZH2, EED, or SUZ12 leads to early embryonic lethality due to the embryos failing to undergo gastrulation [50,51,52]. Studies of conditional and tissue-specific knockout mice demonstrate the importance of PRC2 in orchestrating cellular differentiation in a range of tissues. PRC2 maintains the integrity of cellular identity and loss of essential component proteins makes differentiation more labile [21,53,54]. During embryogenesis, H3K27me3 patterns emerge during the morulae stage and reach their peak in blastocytes [55]. H3K27me3 is widely distributed in a non-canonical pattern among intergenic spacers and gene deserts [56,57]. After fertilization, the paternal H3K27me3 mark is largely removed from the promoter regions of development-related genes, while the maternal mark retained. This allele-specific differential H3K27me3 modification persists till the blastocyst stages [55,58].

Methylation of H3K27 is an important mechanism in the maintenance of self-renewing embryonic stem cells (ESCs) by repressing the differentiation pathway. For instance, a decrease in H3K27me3 levels is seen at the promoter of *UTX* (Ubiquitously Transcribed Tetratricopeptide Repeat on chromosome X) during the differentiation of wild-type ESCs. Additionally, epigenomic analyses suggest that ESCs possess genes with promoters that have abundant H3K27me3 along with a high level of transcription-activating H3K4me3. In these cases, the presence of H3K27me3 at the transcription start site results in transcriptional repression. However, such domains, termed “bivalent” are poised for rapid transcriptional activation facilitating differentiation towards diverse cell fates. Not only are genes with bivalent domains enriched in ESCs [59], they are also enriched in cancer stem cells which also possess the ability to differentiate into multiple cell fates [60].

## 3. Alterations in Inducers of Methylation of H3K27 in Cancer

The methylation of H3K27 is a critical contributor to transcriptional repression in many biological processes including genomic imprinting, X-chromosome inactivation in females, stem cell maintenance and differentiation, circadian rhythm, and especially cancer [61,62]. Not surprisingly, loss of homeostasis in this pathway has emerged as a recurrent theme in the pathogenesis of many cancers.

Epigenetic regulators, considering their importance during normal stem cell maintenance, are typically hijacked to sustain malignant properties. The preponderance of evidence suggests that EZH2 acts as an oncogene and its aberrant over-expression and deregulation of function are documented across multiple varieties of solid tumors, including breast, ovarian, pancreas, prostate, bladder, renal, brain, kidney, gastric, and lung malignancies [23,63,64,65,66,67,68,69,70,71,72,73] (Table 1). Additionally, EZH2 overexpression has been shown to promote migration, cell proliferation and invasion in different in vitro cancer models [74,75,76]. Pointing to a direct role of increased H3K27me3 in the oncogenic capacity of EZH2, the oncogenicity of EZH2, in in vivo models, correlates with its H3K27 methyltransferase activity [77]. Complicating the story, both gain- and loss-of-function mutations in EZH2 are frequent in various types of cancer including lymphomas, melanoma, and myelodysplastic lymphoma (MDS) [22,78]. The gain-of-function mutations in EZH2 often lead to a hyperactive methyltransferase enzyme, resulting in extensive H3K27 trimethylation, promoting chromatin restriction and blocking cell differentiation [62].

**Table 1:** EZH2 alterations by cancer type. Data on expression status was collected from studies deposited in Oncomine (http://www.oncomine.org/) or in the scientific literature. Data on mutation, gene amplification, and deletion were collected from the TCGA PanCancer dataset using cBioPortal [79,80]. Cancer types with alteration at EZH2 in at least 2% of cases are shown. All mutations within the SET domain or truncating mutations prior to the SET domain were considered to compromise enzymatic activity.

*EZH2*						
Cancer Types with Evidence of Altered Expression or with Gene Altered in at Least 2% of Cases	Altered Expression	Cases in TCGA PAN-CANCER				
		Amplification	Fusion	Deep Deletion	Mutation	% of Mutations Affecting the SET Domain
Prostate Adenocarcinoma	↑ [64,66,81]	0.80%	-	0.20%	-	-
Invasive Breast Carcinoma	↑ TCGA [82]	0.65%	-	0.46%	0.28%	100%
Colon Adenocarcinoma	↑ [83,84]	-	-	-	1.85%	17%
Hepatocellular Carcinoma	↑ [85]	0.81%	-	0.54%	0.27%	100%
Endometrial Carcinoma	↑ [69]	0.50%	-	-	7.50%	31%
Clear Cell Renal Carcinoma	↑ [86]	0.59%	-	-	0.39%	0%
Ovarian Epithelial	↑ [TCGA] [87]	7.00%	-	0.30%	0.30%	0%
Melanoma	↑ [69]	2.00%	-	0.20%	4.70%	21%
Sarcoma	↑ [88,89]	1.96%	-	2.35%	0.39%	100%
Cervical Carcinoma	↑ [90,91,92]	-	-	-	4.35%	0%
Lung Adenocarcinoma	↑ [93]	1.04%	0.28%	0.38%	1.61%	0%
Esophagogastric Adenocarcinoma		0.19%	-	1.56%	1.17%	0%
Glioblastoma	↑ TCGA ↓ [94]	1.86%	-	-	0.68%	25%
Leukemia	↑ [95] ↓CLL [96,97,98]	-	-	1.00%	1.00%	25%
Bladder Urothelial Carcinoma	↑ [99]	0.49%	-	0.49%	1.46%	40%
Adrenocortical Carcinoma	↑ [100]	1.10%	-	1.10%	-	-
Esophageal Squamous Cell Carcinoma	↑ [101]	1.05%	-	1.05%	-	-
Mature B-Cell Neoplasms		-	-	-	2.08%	0%
Cholangiocarcinoma		-	-	-	2.78%	0%

In hematological malignancies, the role of EZH2 may be either oncogenic or tumor suppressive [102,103]. The over-expression of mutant EZH2 has been reported in a wide variety of B-and T-cell lymphoproliferative disorders [103,104,105,106]. In chronic lymphocytic leukemia (CLL), studies into the role of PRC2 components in hematopoietic stem cells (HSCs) have been somewhat contradictory. While gain-of function studies by Kamminga et al. and Herrera-Merchan et al. suggest it has an important role in self-renewal regulation [95,107], knockout of *EZH2* in mice by O’Carroll et al. demonstrated no effect on HSCs, although development of B and T cells was affected [50]. While the reason for these conflicting results remains to be determined, some evidence shows that in adult HSCs, EZH1 can compensate for EZH2 loss, which can help to explain these discrepancies [108]. Although many studies use cell lines, primary cells and mouse models of CML have shown dysregulated expression, but no mutations in the PRC2 components [102]. In MDS and MDS-derived acute myeloid leukemia (AML), EZH2 over-expression is correlated with poor prognosis. Xu et al. describe that MDS and MDS-derived AML harbor a specific DNA methylation of the tumor suppressor gene *INK4B* (p15) [109]. Patients with methylation at this gene have statistically higher relative mean expression of EZH2 compared with patients without methylated *INK4B*. According to International Prognostic Scoring System, the level of EZH2 expression is positively correlated with poor disease outcome [103,109,110]. The over-expression of EZH2 is not enough to cause leukemia, but it does prevent the clearance of HSCs [95]. Overexpression of EZH2 may also be caused by a variety of transcriptional pathways, some of them are specific to particular malignancies, while others are more common.

EZH2 has a well-described oncogenic function in solid cancers. In triple-negative breast cancer overexpression of EZH2 is found to be a result of MEK-ERK-ELK1 pathway activation. Phosphorylated ELK1 binds three ELK1-binding motifs within the *EZH2* promoter and activates *EZH2* transcription. In bladder cancer, small-cell lung cancer and breast malignancies, EZH2 dysregulation is related to the deregulation of pRb-E2F pathway or overexpression of E2F [27,111,112,113]. Additionally, MYC and ETS transcription factors in prostate cancer [113,114], NF-YA in epithelial ovarian cancer [115], and STAT3 in colorectal cancer [23] regulate EZH2 expression, while the fusion oncoprotein EWS-FLI1 induces EZH2 expression in Ewing’s sarcoma [116]. Hypoxia in solid tumors can be another potential regulator of EZH2 transcription. Chang et al. identified a consensus sequence for hypoxia-inducible factor-1α (HIF-1α) within the *EZH2* promoter [117]. Along with these signaling pathways, EZH2 expression can be controlled post-transcriptionally by multiple micro-RNAs (miR-25, -26a, -30d, - 144, -214, -138, -137, let-7, etc.). These miRs can downregulate EZH2 by interacting with its 3’UTR sequence, thus, dysregulation of these miRs can up-regulate the EZH2 expression driving cancer progression [118,119,120,121,122,123,124,125,126,127,128,129]. An increase in H3K27me3 can also be the result of increased recruitment of PRC2 complex to chromatin. Long non-coding RNAs (lncRNAs) are one of the potential factors in this regard. In particular, HOTAIR along with several other lncRNAs such as HEIH, PCAT-1, and H19 etc. [130,131,132,133,134,135], play oncogenic roles by interacting with PRC2 to recruit EZH2 to target genes in various cancers.

Altered EZH2 activity may also be due to gain-of-function (GOF) mutations. In their landmark study, Morin et al. describe GOF point mutations resulting from a switch from tyrosine to histidine at codon 641 which lies in the catalytically active SET domain of the EZH2 protein [104]. This study demonstrated that Tyr641 (Y641) mutations (Y641F, Y641N, Y641S, Y641C and Y641H) [22], while associated with significant reduction of EZH2 enzymatic activity, nevertheless resulted in an increase in global H3K27me3, possibly due to a change in substrate preference to favor H3K27me2 over H3K27me0/1 [103,136,137]. The oncogenic potential of this mutation is well established and has been found in 7.2% of follicular lymphoma and 21.7% of diffuse large B-cell lymphoma (DLBCL) [106,138,139,140]. Additional support for an oncogenic role for EZH2 in cancer comes from the loss-of-function mutations of other cancer associated chromatin regulators that normally antagonize EZH2 activity. In malignant rhabdoid tumors, which are highly aggressive pediatric cancers, loss of the SWI-SNF core subunit SNF5 (also known as SMARCB1) results in unopposed EZH2 activity [22,141,142]. Studies from different laboratories extend this antagonistic model in different cancers. Mutation in the SWI-SNF subunit ARIDIA causes synthetic lethality during the inhibition of EZH2 in ovarian cancer xenograft. Furthermore, inhibition of EZH2 in lung cancer xenograft having SWI-SNF subunit BRG1 mutation sensitizes to chemotherapy [143,144]. In addition to methylation of H3K27, EZH2 has been shown to possess oncogenic activity through its ability to methylate cellular proteins and act as a co-activator of steroid hormone receptors. This modification requires an intact methyltransferase domain and phosphorylation of EZH2 [145]. This function is hypothesized to be independent of PRC2 and potentially induced by phosphorylation of EZH2 [145,146].

Although the frequency of overexpression and gain-of-function mutations suggests an oncogenic role for EZH2, there is also evidence suggesting that EZH2 acts as a tumor suppressor in some cancers [22,147,148,149]. Both monoallelic and biallelic mutations (missense mutation, frameshift mutation, premature stop codons and splicesomal mutations) of *EZH2* have been identified in around 23% of various subtypes of MDS, myeloproliferative neoplasms (MPN), and MDS-MPN overlap disorders [150,151,152,153] (Table 1). Almost all mutations are predicated to inactivate the methyltransferase activity. Individuals having homozygous mutations have reduced survival compared to those having heterozygous mutations. Furthermore, loss-of-function mutations and deletions of *EZH2* are also detectable in other cancers including T-lineage acute lymphoblastic leukemia (T-ALL) and core binding factor acute myeloid leukemia. Notably, EZH2 is not the only PRC2 subunit affected in these cancers, as missense mutations have also been identified in SUZ12 [152,154,155].

Clearly, most tumor types use EZH2 in support of tumor growth, yet EZH2 loss also occurs, particularly in some myelodysplastic syndromes, and mutations can drive neomorphic activity, particularly in lymphomas. Still, not all blood cancers lose or alter EZH2 activity. Whether frequency of co-mutations, effects on protein localization, or other alterations in the PRC2 complex members can explain these disparities remains to be seen.

## 4. KDM6 Family-Molecular Functions

Histone lysine methylation was long considered a stable covalent modification due to its low turnover rate relative to other post-translational modifications [30,31]. Nevertheless, two proteins with known demethylase activity can remove H3K27me3: KDM6A, previously known as ubiquitously transcribed tetratricopeptide repeat X (UTX) and KMD6B, previously known as Jumonji domain-containing protein D3 (JMJD3) [156,157,158,159,160]. KDM6A consists of 1401 amino acids and contains a JmjC catalytic domain and 6 tetratricopeptide repeat (TPR) protein-protein interaction domains. KDM6B consists of 1679 amino acids, with only a JmjC domain characterized [159,161]. However, sequence analysis suggests that it may also contain TPR domains like KDM6A [162,163]. A third member of this protein family, KDM6C, which was previously known as UTY, consists of 1347 amino acids, has 83% amino acid homology with KDM6A and was once thought to lack demethylase activity [157,162,164]. However, recently it has been demonstrated that it has minimal demethylase activity due to a subtle sequence diversity in the JmjC domain [164]. *KDM6C* is located on the Y chromosome and, as shown in a knock-out mouse model, it can partially compensate for some of the demethylase-independent functions of KDM6A [165]. KDM6C may activate transcription in a gene-specific manner and it is thought that this demethylase may be required for male sex determination during embryonic development [162,164]. Furthermore, KDM6A is distinct from KDM6B in a functional sense in that its activity is more dependent upon O_2_ concentration [166]. Indeed, hypoxia induces an increase in H3K27me3 which is mediated by loss of KDM6A activity, independent of the canonical HIF-1α response [166].

It has been established that KDM6A associates with KMT2-related H3K4 methylating proteins in the Compass-like complex (Figure 1). In 2007, an KMT22 complex was identified in different mammalian cell types containing 12 protein members including KMT22, PTIP, UTX/KDM6A, RBBP5, WDR5, DPY30, ASC-2, ASHL2L, martin 3, MGC4606, alpha- and beta-tubulin [167]. Furthermore, KDM6A, KMT22 and PTIP co-localize at the promoter regions and first exon of KMT22 target genes, which is marked with H3K4me3 [167]. Another group confirmed a similar H3K4me3 methyltransferase complex containing KMT22, KMT23, KDM6A, PTIP, ASC-2, PAI, ASH2L, RBBP5, DPY30, and WDR5 [168]. Interestingly, the composition of this KMT22 complex was conserved with Drosophila ortholog Trithorax related (trr) complex [169] and *C. elegans* ortholog SET-16 complex [170]. This finding suggests a tight coupling in the reciprocal relationship of H3K4 and H3K27 tri-methylation during transcriptional gene regulation [61,167,171]. Although aspects of their biochemical regulation still remain to be determined, demethylase enzymes like KDM6A and KDM6B are often genetically inactivated in various types of cancers [7,172] (Table 2 and Table 3). Therefore, it is reasonable to hypothesize that homeostasis between EZH2 and KDM6A/B activity is necessary for maintaining normal cell activity or phenotype (Figure 1).

## 5. Alterations in the Erasers of Methylation of H3K27 in Cancer

Depending on the cell or cancer type KDM6 family members have both oncogenic and tumor-suppressor roles in cancer [61,173,174,175,176]. KDM6A was initially characterized as a tumor-suppressor, which can repress Notch and Rb-induced tumors in Drosophila [173,177]. Interaction between Rb and KDM6A was also demonstrated in human cells. Loss-of function mutations and deletions targeting *KDM6A* have been reported in medulloblastoma [178], T-cell acute lymphoblastic leukemia (T-ALL) [175,176], acute lymphoblastic leukemia (ALL) [175,179], renal cell carcinoma [180], bladder cancer [181,182], chronic myelomonocytic leukemia (CMML) [153], multiple melanoma [183], and many other solid and non-solid tumors [183] (Table 2).

**Table 2:** KDM6A alterations by cancer type. Data on expression status was collected from studies deposited in Oncomine (http://www.oncomine.org/) or in the scientific literature. Data on mutation, gene amplification, and deletion were collected from the TCGA PanCancer dataset using cBioPortal [79,80]. Cancer types with alteration at KDM6A in at least 2% of cases are shown. All mutations within the JmjC domain or truncating mutations prior to the JmjC domain were considered to compromise enzymatic activity.

*KDM6A*						
Cancer Types with Evidence of Altered Expression or with Gene Altered in at Least 2% of Cases	Altered Expression	Cases in TCGA PAN-CANCER				
		Amplification	Fusion	Deep Deletion	Mutation	% of Mutations Affecting the JmjC Domain
Bladder Urothelial Carcinoma	↑ [184]	0.49%	-	2.68%	25.55%	74%
Esophageal Squamous Cell Carcinoma	↓ [101]	1.05%	-	11.58%	3.16%	25%
Non-Small Cell Lung Cancer		0.85%	0.09%	2.18%	3.23%	49%
Endometrial Carcinoma		0.85%	-	0.68%	6.66%	22%
Head and Neck Squamous Cell Carcinoma	↓ [185]	0.19%	-	3.25%	3.44%	65%
Pancreatic Adenocarcinoma		-	-	2.72%	3.80%	86%
Stomach Adenocarcinoma		0.68%	-	1.82%	3.41%	40%
Melanoma	↓ [186]	-	-	0.23%	5.18%	11%
Cervical Squamous Cell Carcinoma		0.34%	-	1.35%	3.03%	40%
Prostate Adenocarcinoma	↑ [187]	0.20%	-	1.21%	2.02%	25%
Mature B-Cell Neoplasms		2.08%	-	-	2.08%	0%
Ovarian Epithelial Tumor	↓ [188]	2.23%	0.17%	1.03%	0.68%	33%
Lung Adenocarcinoma		0.88%	0.18%	0.71%	1.94%	31%
Esophagogastric Adenocarcinoma		0.97%	-	1.95%	3.50%	0%
Colorectal Adenocarcinoma		0.17%	0.51%	-	2.53%	33%
Renal Non-Clear Cell Carcinoma		-	-	-	3.16%	0%
Sarcoma		1.18%	-	0.78%	0.39%	25%
Hepatocellular Carcinoma		0.54%	-	1.63%	0.81%	33%
Invasive Breast Carcinoma	↑ [189]	0.65%	0.09%	0.18%	1.94%	0%
Leukemia	↑ Adult T-cell [190]	0.50%	-	0.50%	1.50%	0%
Pleural Mesothelioma		-	-	1.15%	1.15%	0%
Cervical Adenocarcinoma		-	-	-	2.17%	29%

Genetic loss of *KDM6A*, co-occurring with aberrant TLX3 expression in T-ALL, accelerates the initiation and progression of leukemia and decreases the life span in NOTCH1-overexpressing experimental mice [175,176,191]. Overexpression of KDM6A also decreases growth and induces apoptosis in T-ALL cell lines [176]. Tying this result to its H3K27me3 demethylase activity, loss of KDM6A leads to accumulation of H3K27me3 at the promoters of putative tumor suppresser genes, including *FBXW7* and *RBBP6* [173,175]. Additionally, the effects of KDM6A knockdown are also reported to be dependent on EZH2 activity as they can be blocked by the EZH2 inhibitor 3-DZNep [192].

Surprisingly, KDM6B, in contrast to KDM6A, acts as an oncogene in NOTCH1 overexpressing T-ALL cancer (Table 3) [192]. ChIP studies reveal that KDM6B is bound to NOTCH1 targets with oncogenic functions (HEY1, NRARP and HES1). Indeed, knockdown of KDM6B affects the viability of leukemic cells. Consistent with its function as a transcription activator, genome-wide analysis shows that more transcripts are significantly downregulated than are upregulated by KDM6B knockdown. Nevertheless, one hypothesis that may explain the oncogenic function of KDM6B in T-ALL, versus the tumor suppressive function of KDM6A in T-ALL, could be the lack of N-terminal TPR domain in KDM6B, but the exact cause and mechanism is yet to be explored [175,193].

**Table 3:** KDM6B alterations by cancer type. Data on expression status was collected from studies deposited in Oncomine (http://www.oncomine.org/) or in the scientific literature. Data on mutation, gene amplification, and deletion were collected from the TCGA PanCancer dataset using cBioPortal [79,80]. Cancer types with alteration at KDM6A in at least 2% of cases are shown. All mutations within the JmjC domain or truncating mutations prior to the JmjC domain were considered to compromise enzymatic activity.

*KDM6B*						
Cancer Types with Evidence of Altered Expression or with Gene Altered in at Least 2% of Cases	Altered Expression	Cases in TCGA PAN-CANCER				
		Amplification	Fusion	Deep Deletion	Mutation	% of Mutations Affecting the JmjC Domain
Papillary Renal Cell	↑ [194]	-	-	0.35%	1.06%	66%
Oligodendroglioma	↑ [195]	-	-	-	-	-
Melanoma		-	-	0.45%	8.33%	13%
Endometrial carcinoma		0.17%	-	0.17%	7.68%	19%
Miscellaneous Neuroepithelial Tumor		-	-	3.23%	3.23%	0%
Colorectal Adenocarcinoma		-	-	1.18%	4.21%	38%
Esophagogastric Adenocarcinoma		0.19%	-	0.58%	4.09%	0%
Prostate Adenocarcinoma	↑ [196]	-	-	4.05%	0.81%	25%
Esophageal Squamous Cell Carcinoma		1.05%	-	1.05%	2.11%	50%
Sarcoma		1.57%	-	1.18%	0.78%	0%
Adrenocortical Carcinoma		-	-	1.10%	2.20%	0%
Hepatocellular Carcinoma	↑ [197]	-	-	2.44%	0.54%	0%
Non-Small Cell Lung Cancer		0.09%	-	0.76%	1.99%	0%
Bladder Urothelial Carcinoma		-	-	0.97%	1.70%	14%
Cervical Squamous Cell Carcinoma		-	-	0.40%	1.99%	0%
Cervical Adenocarcinoma		-	-	2.17%	-	-
T-Cell Lymphomas	↓ [198]	-	-	-	-	-
Mature B-Cell Neoplasms	↓ [97]	-	-	2.08%	-	-

KDM6A is not a tumor suppressor in all T-ALL molecular subtypes. In cases of T-ALL that are driven by transcription factor TAL1, KDM6A acts as a coactivator for oncogenic reprogramming [192]. In myeloid leukemia, suppression by KDM6A depends on its non-catalytic activity. KDM6A helps to suppress the effect of ETS (E-twenty-six), while supporting the activation of those targeted by GATA transcriptional programs, mainly driven by GATA2 [199]. These effects mainly impact the enhancers [200].

Bladder cancer has one of the highest frequencies of KDM6A mutations, which are typically truncating mutations compromising the catalytic JmjC domain. Interestingly, in almost 50% of bladder cancer cases there is a significant genetic aberration in chromatin remodeling genes, such as *KMT2C* (19.7%), *KMT2D* (28.3%), *NCOR1*, *EP300* (16%), and *CREBBP* (13.3%) along with *KDM6A* [182]. Whole genome sequencing reveals that, in almost 25% cases, KDM6A is inactive [181,200,201,202]. There are divergent reports of KDM6A’s role in breast cancer. Wang et al. describe that low KDM6A expression predicts poor survival in breast cancer [203], while Kim JH et al. report that high expression is associated with poor prognosis [204]. However, later studies support an oncogenic role for KDM6A as it is overexpressed in breast cancer and correlates with cancer grades [189,205]. Furthermore, knockdown of KDM6A decreases cancer cell proliferation, invasion, and lung colonization [162,189]. In breast cancer as well as others, the confusion concerning KDM6A’s role may be attributed to its suite of protein-protein interaction (KMT2D vs LSD1/HDAC1) or whether the activity is demethylase-dependent or demethylase-independent [63].

KDM6B can also serve as a tumor suppressor or oncogene. It may act as a tumor suppressor by binding to and activating the INK4A-ARF locus [197]. When cells are exposed to oncogenic stress, the H3K27 residues at this locus can become demethylated, leading to cell cycle arrest and senescence [206,207]. Furthermore, *KDM6B* is located on chromosome 17 in close proximity to tumor suppressor *TP53*. Functional interaction between KDM6B-p53 and resulting p16 activation is well documented and deletions involving both genes are commonly found in human cancers [173,208,209]. On the other hand, KDM6B appears to act as an oncogene in melanoma and B-cell lymphoma [162]. Depletion of KDM6B in melanoma induces self-renewal, invasive migration, metastasis and angiogenesis [210]. Similarly, knockdown of KDM6B reduces tumor growth and induces apoptosis in diffuse large B-cell lymphoma [211]. In hepatocellular carcinoma, KDM6B is highly expressed and positively corelated with distant metastasis, tumor diameter, vascular invasion, differentiation, and poor prognoses in patients [212]. It has also been reported that KDM6B and HER2 contribute to poor prognoses of ovarian cancer by inducing cell migration and invasion through the regulation of TGF-β1 [213,214]. Upregulation of KDM6B also positively corelates with poor prognosis in gastric cancer [215]. In acute myeloid leukemia (AML) KDM6B is upregulated and correlated with poor prognosis [216]. In colon cancer, glioma cells, and esophageal squamous carcinoma KDM6B is upregulated [217,218,219,220].

Similarly, mutations (nonsense mutation, frameshift mutation, deletion, insertion etc.) in *KDM6A* in different cancers (renal, pancreatic, bladder, esophageal, myeloma etc.) cause the premature termination of codons and results in the loss of demethylase activity. Consequently, the loss-of-function mutations in KDM6A probably has effects analogous to EZH2 gain-of-function mutations [153,182,183,202,221].

Whether H3K27me3 demethylation is a trick or treat in cancer is highly contextual. The disparate effects caused by LOF of either KDM6A or KDM6B indicate a complex relationship between the molecular biology of H3K27me3 removal and the cellular biology of cancer development. A clearer understanding of the factors regulating recruitment and activity of each of these enzymes within the Compass-like complex and to genes regulating distinct functions is needed to further understand the mechanistic basis of these observations.

## 6. Alterations in the Substrate and Readers of Methylation of H3K27 and Alterations in DNA Methylation in Cancer

In a set of landmark findings, genome sequencing efforts from two different groups, Schwartzentruber et al. [222] and Wu et al. [223], demonstrated a high frequency of mutations in H3F3A (>70%, one of two genes encoding H3.3 [224]) converting lysine 27 to methionine (H3K27M) in pediatric high-grade glioma (pHGG) and that this mutation is an indicator of poor survival. Further studies from different laboratories extended the list of cancers known to carry mutations in H3 to include chondroblastoma, giant cell tumors in bone, chondrosarcoma, pediatric soft tissue sarcoma, head and neck squamous cell sarcoma, and leukemia [225,226,227,228,229,230]. Intriguingly, H3K27M mutations are not detected in other pediatric brain tumors such as medulloblastomas and ependymomas and rather seems to be specific for midline pHGGs [224,231].

Histone 3 has different variants, among which H3.3 is expressed constitutively [232,233,234]. These substitutions in the core histone protein dictate the binding specificity of the chaperone proteins, control their incorporation into and eviction from nucleosomes, thus contributing to differential localization of the H3 variants in the genome [235,236,237,238]. H3.3 is enriched at pericentromeric and telomeric regions and, importantly, at transcriptionally “active” or “poised” regions [239,240,241]. These H3.3-marked, poised, regions are also enriched for H3K4me3 or are bivalent: containing both H3K4me3 and H3K27me3. Therefore, deposition of any mutated histone protein can disrupt the homeostasis of bivalent domains [242]. *In vitro* evidence also suggests that H3K27M mutations act as a dominant negative inhibitor by disrupting PRC2 activity [236,243]. Bender et al. [236,243] and Chan et al. [244] established that mutant histones can disrupt the transcriptional program by ‘trapping’ the PRC2 complex, preventing deposition of the methyl mark on other PRC2 target regions, a process also linked to DNA hypomethylation. Funato and colleagues demonstrated that H3K27M synergizes with p53 loss and PDGFRA activation to induce DIPG (disuse intrinsic pontine glioma) [245]. Additionally, DNA methylation and H3.3K27M can work in concert to stabilize the tumor phenotype [45,63,236,243,246,247,248].

Apart from the altered expression of or mutations in H3K27 writers (methyltransferases) and erasers (demethylases), alterations in readers and dysregulation of other chromatin regulators are also tied to H3K27me3 regulation. Epigenetic information, marked in histone structures, is decoded by these reader proteins, which can bind to chromatin marks using specific structures, such as chromodomains and PHD domains [249]. Alterations in the readers can mis-transduce the signal, thereby altering the functional outcome of the signal. The chromodomain of reader proteins recognizes lysine methylation marks in histones and other proteins. These proteins can recruit effector molecules and propagate and maintain a silent chromatin conformation with the help of WD40 domains [63]. Loss of heterozygosity for the chromodomain protein CDYL is found in cervical cancers and causes the de-repression of the proto-oncogene *NTRK3*, associated with poor prognosis [250]. Chromobox Homolog (CBX) proteins are responsible for recruiting the PRC1 complex at specific loci by recognizing H3K27me3 marks. CDYL binds H3K27me3 and recruits EZH2, bridging the PRC2 complex with already modified chromatin [251]. Loss of CBX7 expression is associated with invasiveness and EMT in many cancer types [252,253,254]. However, in leukemia, overexpression of CBX7 in hematopoietic stem progenitor cells enhances self-renewal, thus acting as an oncogene [255]. Mutation in EED seems to disrupt the formation of the PRC2 complex and abrogate the methyltransferase activity [256,257]. One rare mutation in the WD40 motif of EED can inhibit its interaction with H3K27me3, and therefore allow for the global H3K27me3 level to decrease [63,258].

Many histone modifying enzymes can also indirectly impact H3K27me3, either at specific loci or genome wide. In cases of multiple myeloma (MM) bearing the t(4;14) translocation, *MMSET* is fused to the immunoglobin heavy chain locus, leading to its overexpression. Overexpression of MMSET causes a genome wide increase in abundance of H3K36me2 and a global reduction of H3K27me3 [259,260,261]. Nevertheless, Popovic et al. describe how, in melanoma, there are a few loci which are shielded from this MMSET overexpression and can exhibit a high level of H3K27me3 and thus repression of transcription of such specific loci [262]. A similar interplay was observed in B-cell ALL (B-ALL) and mantle cell lymphoma, where a recurrent mutation in SET domain of MMSET mimics the effects of MMSET overexpression [263,264]. In AML, there is a t(5;11) translocation, which leads to fusion of MMSET homolog NSD1 and NUP98, resulting in loss of EZH2 and H3K27me3 at the HOXA locus [246].

Alterations in chromatin-remodeling complex proteins or other histone modifying proteins can also affect H3K27me3. Loss of SNF5, part of the SWI/SNF chromatin-remodeling complex, decreases polycomb protein displacement at specific loci, leading to an increase in EZH2 and H3K27me3 in T-cell lymphoma [142,265]. ARID1A of the SWI/SNF complex, is frequently mutated in many tumors. In ovarian clear cell carcinoma (OCCC) ARID1A mutation selectively suppresses cell proliferation by reducing H3K27me3 in selective loci [143,266,267]. HDACs (HDAC1 and HDAC3) interact with PRC2 and promote H3K27 methylation by removing the acetylation on H3K27, antagonizing the effect of CBP/p300. HDAC expression is increased in various cancers and therefore impacts H3K27me3 levels [268,269,270].

Wilms’ tumor 1 (WT1) is a transcription factor which can interact with DNA-demethylating enzymes. In about 10% of AML cases, the *WT1* gene harbors an inactivating mutation within its DNA-binding zinc finger domain, leading to an increase in DNA methylation. Hypermethylation then overlaps with genes targeted by PRC2 to increase H3K27me3, resulting in strong suppression [271,272,273]. An H3K4-specific KMT, KMT2 (KMT21), is frequently rearranged in AML along with a large number of other genes [274]. Although, due to fusions, KMT2 typically loses its KMT activity, it can still recruit coactivators of transcription and epigenetic modifiers, like EZH2, causing a disruption of the “bivalent” state [275]. It has been well established that monoubiquitinated H2AK119 (H2AK119ub1) can recruit the PRC2 complex to maintain chromatin repression [276,277]. In around 40% of breast cancer cases, TRIM37, a ubiquitin ligase for H2AK119ub1 is amplified resulting in higher recruitment of PRC2 and higher levels of H3K27me3 correlating with decreased survival of ER+ patients [63,277]. Overall, aberrations in readers, mutations in H3 itself, and other chromatin regulators of H3K27me3 can contribute outcomes as dramatic as ones caused by deregulation of chromatin writers and erasers.

Belying the common view that H3K27me3 is oncogenic, mutations of this residue within histone-coding genes have now been shown as oncogenic in some cancers. Nevertheless, reader proteins are either lost or over-expressed in multiple cancers further illustrating the context-specific interaction between H3K27me3 and cancer.

Aberrant 5mC contributes to tumor progression and cancer-drug resistance, concepts reviewed recently by Göndör and colleagues [5]. In particular, a distinct subset of 5mC-driven cancers, termed CpG island methylator phenotypes (CIMPs), shows hypermethylation of a subset of CpG islands [278,279]. Later research made possible by the TCGA project identified CIMPs in colorectal, breast, and endometrial tumors as well as in glioblastomas and acute myeloid leukemias, but not in lung squamous, kidney renal cell cancers or serous ovarian cancers [280]. In cancers with global hypomethylation, however, the activity of PRC2 is expanded and contributes to gene silencing [281].

In glioblastoma multiforme (GBM) Parsons and Colleagues identified specific heterozygous somatic point mutations in the isocitrate dehydrogenase 1 (IDH1) most often in R132 [282]. In 2010, Noushmer and TCGA network colleagues showed an extremely tight correlation between *IDH1*R132H mutation and glioma CIMP (G-CIMP), although there were also a small number of WT *IDH1* (*IDH1*WT) G-CIMP tumors [283,284]. According to TCGA database G-CIMP tumors are tightly associated with proneural subgroup. Further studies shown that introduction of *IDH1*R132H in cell lines with endogenous wild type *IDH1* was sufficient to drive G-CIMP based DNA methylation events and increase the prevalence of H3K9me2, H3K27me3 and H3K36me3 [285,286]. These epigenomic changes mainly occur through changes of metabolic function of IDH proteins. Normally, IDH catalyzes the reduction of NADP+ (nicotinamide adenine dinucleotide phosphate) to NADPH by converting isocitrate to α-ketoglutarate (α-KG) [287]. However, mutant IDH catalyzes the conversion of α-KG to D-2-hydroxyglutamate (2-HG) [288,289,290,291,292]. Since 2-HG inhibits both the Jumanji-C domain containing histone lysine demethylases and the TET family of enzymes [293,294], both pathways for removing methylation may be impacted.

## 7. Cancer Cell Plasticity, EMT, and H3K27me3

Cancer cell plasticity is a subversion of the conserved cellular processes that are signature features of embryonic development. It was traditionally thought that cellular differentiation and developmental options are restricted to a specific lineage fates in higher vertebrates, as opposed to “lower” vertebrates such as amphibians. However, recent experimental evidence indicates that mature mammalian cells are not as stable as was thought and can be induced to dedifferentiate or transdifferentiate. Investigators revealed importance of nuclear regulation to the “hidden plasticity” in differentiated cells by introducing one or more transcription factors [295,296,297,298,299]. In addition to its biological significance, such plasticity also makes possible the emergence of aberrant lineages when the epigenetic mechanisms maintaining cellular identity are subverted. Cancer is one of the possible outcomes of this aberrant reprogramming, whereupon the transcriptional and epigenetic states become disrupted, altering cellular lineages [300].

EMT or epithelial-mesenchymal transition, as its name implies, converts epithelial cells to mesenchymal cells by repressing the genes critical for epithelial states (e.g., E-cadherin, claudins) [301]. EMT can occur in response to normal physiologic signals, including hypoxia, extracellular matrix stiffness [302,303] and transforming growth factor β (TGFβ) [304,305], and is orchestrated by integrated networks of signal transduction pathways, transcription factors (Snail, Twist, Slug, FOXC2, and ZEB1/2 etc.) [306,307,308,309] and microRNAs [310,311,312,313]. EMT and its reverse process, mesenchymal-epithelial transition (MET), are important developmental reprogramming events that are nevertheless strongly implicated in tumor cell invasion and metastasis. In regard to epigenetic regulators, we now have an increased understanding of the roles of nucleosome remodeling complexes [314,315,316], DNA methylation, and de-methylation pathways [60,311], and histone-modification regulating enzymes [317,318,319].

Regulation of H3K27me3 is intimately involved in progression through EMT and MET. The PRC2 complex, by increasing H3K27me3 at target genes, leads to suppression of transcription. Thus, recruitment of PRC2 to the *CDH1* gene, which encodes for E-cadherin, is one mechanism used by EMT transcription factors, in particular Snail1, to induce EMT in pancreatic, gastric, and breast cancer [320,321,322,323]. Using experimental models, expression of Twist was also shown to be sufficient for H3K27me3 accumulation at the *CDH1* promoter [60]. Indeed, an elevated EMT signature, such as found in basal-like or BRCA-mutated breast cancers, correlates with overexpression of EZH2 [117,324,325,326,327]. While lacking enzymatic activity, overexpression of other PRC2 complex members, including BMI1 or EED, also correlates with EMT features and enhanced cancer progression [328,329,330].

Despite the clear association of EZH2 activity with EMT, the suppression of gene expression by H3K27me3 is not restricted to EMT-suppressed genes as EMT-promoting genes are also frequently silenced by H3K27me3. Indeed, in a model of EMT induced by Twist overexpression, a greater number of genes lose H3K27me3 than gain this modification [60]. As a case in point, the *ZEB1* locus is subject to silencing by H3K27me3 in differentiated cancer cells and the modification is removed upon EMT-driven conversion into cancer stem cells [331].

Erasure of H3K27me3, through the activity of KDM6A or KDM6B, also impacts the EMT/MET status of individual cells. Just as both EMT-promoting and EMT-suppressing genes can be silenced by addition of H3K27me3, either set of genes (as well as bivalently modified genes [332]) can conversely be activated by removal of H3K27me3. Illustrating the complexity of epigenetic regulation, KDM6B, like its enzymatic counterpart, EZH2 [333,334], is thought to promote EMT, an apparent paradox explored expertly in the recent review by Lachat et al. [335]. The likely “key” to unlocking this paradox lies in separating broad-based effects, due to changes in expression and activity of these enzymes, from the gene-specific effects driven by recruitment to precise enhancer and promoter regions. This was recently illustrated by Piexoto et al. who, using three models of EMT, used ELISA to demonstrate a general increase in H3K27me3 yet loss of the modification at specific ECM genes including ADAM19 and MMP9 [336].

On the other hand, KDM6A, while also possessing H3K27me3 activity is thought to prevent EMT and promote MET, partially through targeting *CDH1* for activation [337,338,339]. We and others have shown that inhibition of the KDM6 family, through use of the small molecule GSK-J4, increases EMT and stemness features hinting at a dominant role of KDM6A over KDM6B in the governance of cellular states [339,340,341]. Yet, precisely how the two enzymes with identical activity drive disparate outcomes, for instance, by being recruited to distinct target genes, remains to be determined. In invasive breast cancer cell lines, depletion of KDM6A leads to an increase in Myc-dependent EMT factors including SNAI1 and ZEB1/2 [338]. The oxygen-dependent nature of KDM6A is also relevant in cell plasticity as hypoxia-induced loss of KDM6A function leads to suppression of DICER transcription and decreased expression of microRNAs that promote the epithelial state [342]. While these data point to clear mechanistic associations between KDM6A loss and cancer progression, other studies provide strong rationales for therapeutic inhibition of KDM6 proteins in cancer.

Concurrent action of both KDM6A and KDM6B is frequently associated with cell cycle in differentiated or cancer stem cells. In estrogen receptor (ER)-positive breast cancer, KDM6A protein is physically associated with ER and is necessary for the expression of hormone-response and proliferation-inducing genes. Indeed, *in vivo* administration of a dual KDM6- and LSD1-targeted inhibitor, MC3324, suppresses growth of ER-dependent breast cancers [343]. Conversely, KDM6 activity can also be associated with loss of proliferation as entrance of glioblastoma stem cells into a reversible, drug-tolerant, slow-cycling state is dependent on up-regulation and activity of KDM6A and KDM6, which can be prevented by GSK-J4, an inhibitor of both enzymes [344,345].

Considering the strong associations between EZH2, KDM6A, KDM6B and development, the link between these proteins and cancer cell plasticity will continue to become more apparent. Inhibitors of each of these enzymes are efficacious in altering the cancer cell differentiation state, sometimes toward stemness and sometimes toward differentiation, with associated effects on EMT/MET status and drug-resistance. To leverage this axis and improve cancer treatment will require both a better understanding of the intricacies of each molecular player and an improved set of molecular inhibitors, in order to inform carefully constructed pre-clinical experiments.

## 8. Conclusions

The human genome contains several thousand genes, each of which is regulated by proximal and distal elements that cooperate to induce expression of the coding sequences. Multi-genic regions are then organized into topologically associating domains and bordered by insulators which impact gene expression levels in a cell type-specific fashion [8,346,347,348]. Localization and compaction of these loci in the nucleus is a key determining factor for their activity [78,349,350]. Whereas certain stimuli block the differentiation program of cells by increasing the chromatin compactness, others can facilitate the permissive state, thereby inducing the cells’ adaptive responses and can induce oncogenes.

We can conceptualize the permissive state of epigenetic interaction as lowering the height of the walls between canals in Waddington’s landscape [8,351]. This permissive or “plastic” state allows cells to reroute to an alternative transcriptional state, using gene regulation for adaptive responses. When these altered transcriptional states propagate through cellular division, a new “clone” will arise and expand, as in oncogenic propagation. A large number of cancers show marked cell-to-cell variability in gene expression and functional phenotype [352]. Thus, we can hypothesize that epigenetic plasticity is one of the enabling factors that allow premalignant or malignant cells to adopt alternative pathways giving rise to epigenetic “adaptor-clones” that fuel tumor progression.

H3K27me3-regulating proteins are clearly involved in both cellular transformation and in cancer cell plasticity. Leveraging these findings into improved treatments will clearly require distinct plans depending on the tumor type and the predominance of either EZH2 or the KDM6 proteins in supporting tumor growth. An EZH2 inhibitor, tazemetostat, is currently in multiple early phase clinical trials for both solid tumors and lymphomas, in combination with other agents including immunotherapy and chemotherapy [22] (clinicaltrials.gov). Drug development of KDM6-specific inhibitors has not yet reached the level of clinical trial, in part due to the difficulty in achieving true specificity for the KDM6 family and avoiding other JmJC domain containing proteins [353]. Additional medicinal chemistry and drug discovery may facilitate testing of highly-specific inhibitors. Nevertheless, pre-clinical data supporting the applicability of targeting KDM6 proteins continue to emerge [192,354]. As highlighted above, cancer cell plasticity can drive tumor progression, in particular chemotherapy resistance, which can be dependent on epigenetic mechanisms including KDM6 activity [344]. Applying epigenetic-targeting molecules in the context of treatment resistance may enhance therapeutic efficacy in patients with tumor progression.
